# Successful Treatment of Elderly Male With COVID-19 Infection With Severe Acute Respiratory Distress Syndrome Using Multimodal Therapy, Including Immune Modulation Therapy

**DOI:** 10.7759/cureus.12402

**Published:** 2020-12-31

**Authors:** Kei Jitsuiki, Isana Katayama, Toshihide Iida, Setsuko Nagatomo, Youichi Yanagawa

**Affiliations:** 1 Acute Critical Care Medicine, Shizuoka Hospital, Juntendo University, Izunokuni, JPN; 2 Respirology, Shizuoka Hospital, Juntendo University, Izunokuni, JPN; 3 Nursing, Shizuoka Hospital, Juntendo University, Izunokuni, JPN

**Keywords:** covid-19, immune modulation therapy, glycyrrhizin, intravenous immunoglobulin

## Abstract

A 78-year-old man fell from a ladder and suffered a right distal tibial fracture. On the seventh day after injury, he developed a low-grade fever and was isolated in a private room. Polymerase chain reaction for COVID-19 was positive (day 4 from the day of saliva sampling). On day 5, he required 1 liter per minute of oxygen and dexamethasone therapy was initiated. On day 6, his D-dimer level was 25.0 μg/mL, and continuous infusion of heparin was initiated. From day 7, he was administered remdesivir. On day 9, his oxygenation suddenly showed a remarkable deterioration. He received a tentative diagnosis of COVID-19-induced pneumonia accompanied by severe acute respiratory distress syndrome (ARDS) and underwent urgent tracheal intubation and mechanical ventilation. He also received intravenous immunoglobulin (IVIG) and was also administered glycyrrhizin. His oxygenation gradually improved and extubation was performed on day 15. Following rehabilitation, he did not require oxygen on day 19. On day 20, his D-dimer level was found to be increased and enhanced computed tomography revealed pulmonary embolism. He was prescribed a direct oral anticoagulant. On day 28 he was transferred to a general ward for rehabilitation.

These unspecific antiviral therapies and immune modulation therapy may be useful treatments for the main cause of ARDS, which may explain the favorable outcome that was obtained in the present case.

## Introduction

At the time of writing this report, November 2020, the COVID-19 pandemic is ongoing throughout the world. A comparison between Japan and the rest of the world, based on data from the World Health Organization (https://covid19.who.int/, November 24, 2020), revealed that the ratio of confirmed cases is 133929/58712326 (approximately 1/438) and that the mortality ratio is 1989/1388528 (approximately 11/698). Accordingly, the head of the WHO deemed Japan's battle against coronavirus a success (KYODO NEWS - May 26, 2020). However, Japan is in the third wave of the COVID-19 pandemic, and Japan is going into winter when the survivability of coronavirus (SARS-CoV-2) increases due to low temperature and humidity, and it is impossible to predict whether this successful trend will continue into the future [1].

The reported risk factors for mortality in COVID-19 patients are elderly age, male sex, metabolic disease, hypertension, chronic obstructive pulmonary disease, and/or chronic renal failure [2,3]. We herein report a case in which an elderly male patient survived after developing severe acute respiratory distress syndrome (ARDS) requiring mechanical ventilation; the patient had been treated by multimodal therapy, including immune modulation therapy.

## Case presentation

**Figure 1:**
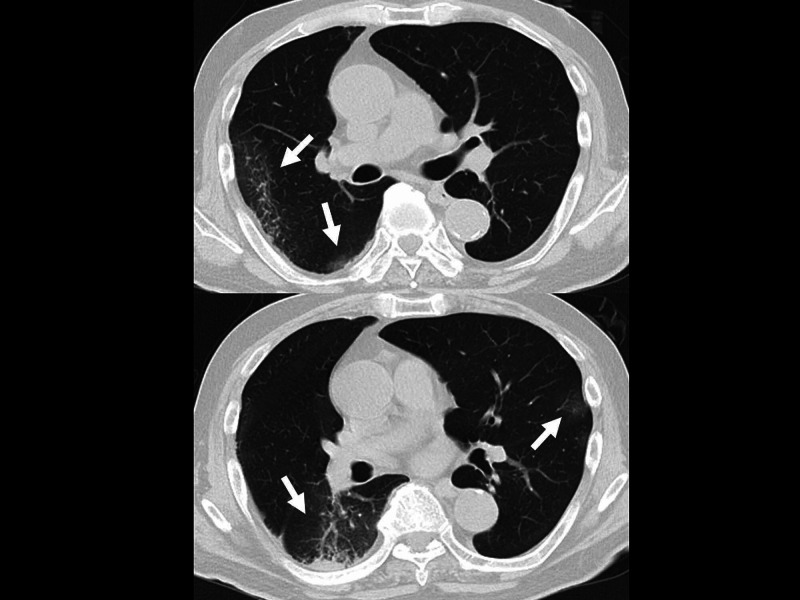
Thoracic computed tomography CT revealed bilateral local peripheral ground-glass opacities (arrow) in the lung fields.

A 78-year-old man fell from a ladder and suffered a right distal tibial fracture. He underwent surgical immobilization on the sixth day after the injury. On the following day, he developed a low-grade fever and was isolated in a private room. Polymerase chain reaction for COVID-19 was positive (day 4 from the day of saliva sampling), and he was moved to an isolated ward for COVID-19 infection. The patient had hypertension, mild chronic pulmonary disease (Brinkman index >1000), left gingival cancer that had been surgically treated, and benign prostatic hypertrophy. He had no specific family history. When a physician checked him, a physical examination revealed the following findings: consciousness, clear; blood pressure, 130/58 mmHg; heart rate, 50 beats per minute; respiratory rate, 20 breaths per minute; SpO_2_, 98% under room air; and body temperature, 38.4 °C, with no specific findings other than those associated with his orthopedic operation. Chest roentgenography revealed no specific findings. Electrocardiography revealed sinus bradycardia. Thoracic computed tomography revealed bilateral local peripheral ground-glass opacities in the lung fields (Figure 1).

**Figure 2:**
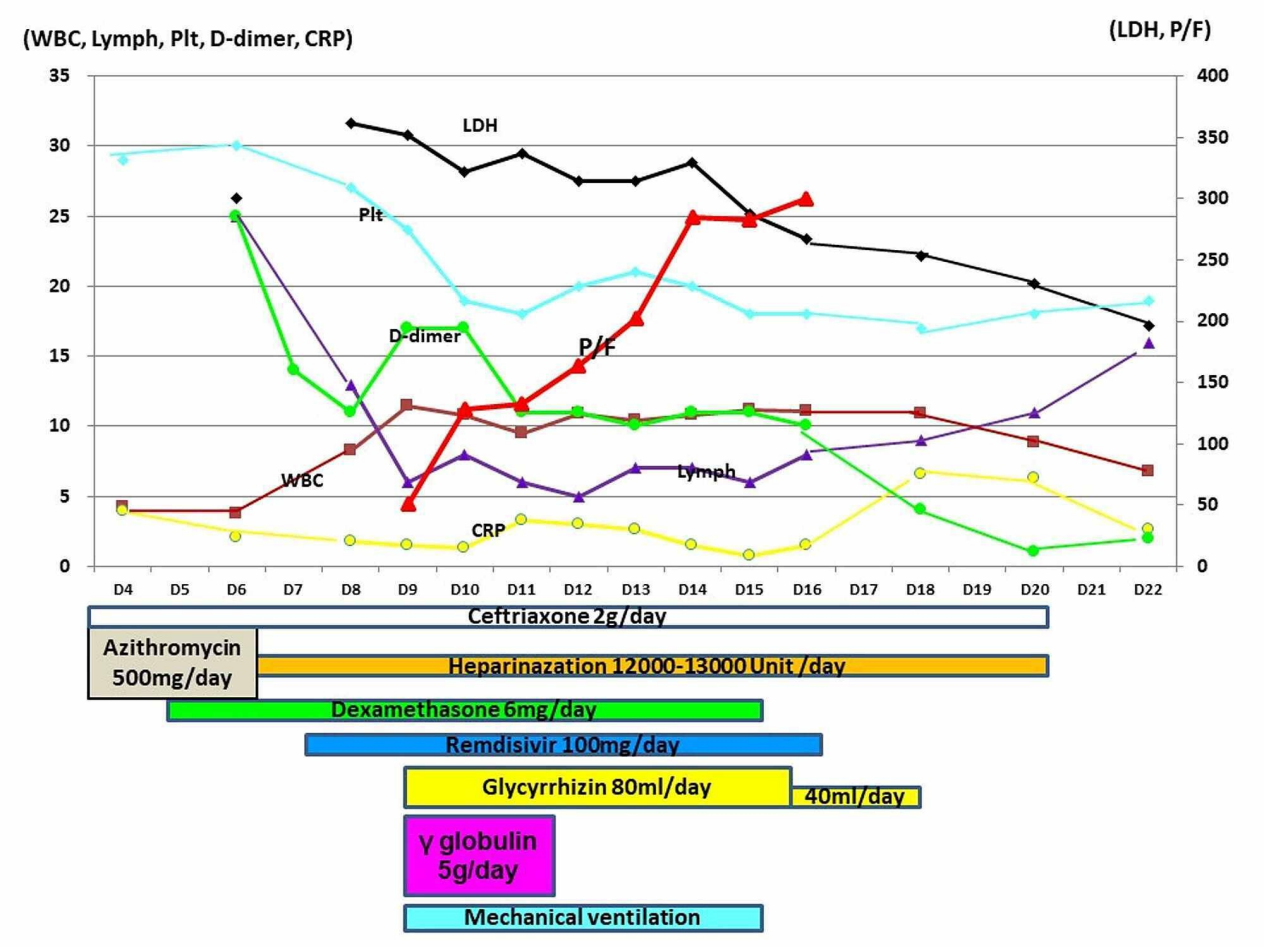
The patient’s clinical course with treatment and the results of a biochemical analysis The PaO_2_/FiO_2_ value improved after the initiation of glycyrrhizin and gamma-globulin therapy.

The main results of a blood analysis were as follows: white blood cell count, 4200/μL; hemoglobin, 11.9 g/dL; platelet count, 30.1×10^4^/μL; glucose, 111 mg/dL; HbA_1_C, 6.6%; total bilirubin, 0.4 mg/dL; aspartate aminotransferase, 81 IU/L; alanine aminotransferase, 59 IU/L; lactate dehydrogenase, 300 IU/L; blood urea nitrogen, 13.2 mg/dL; creatinine, 0.94 mg/dL; creatinine phosphokinase, 119 IU/L; sodium, 138 mEq/L; potassium, 4.5 mEq/L; chloride, 7101 mEq/L; and C-reactive protein, 3.96 mg/dL. As he initially had no oxygen demand, the initial treatment was an infusion of ceftriaxone (2 g/day) and azithromycin (500 mg/day). However, on day 5, he required 1 liter per minute of oxygen (by nasal cannula) to maintain a percutaneous saturated oxygen level of >90%, and dexamethasone (6 mg/day) was prescribed. On day 6, he felt dyspnea on motion and his oxygen demand increased from 1 liter per minute to 2 liters per minute. As his D-dimer level was 25.0 μg/mL, continuous infusion of heparin (12,000 unit/day) was initiated. Infusion of remdesivir (100 mg/day) was initiated from day 7 when the drug was delivered to our hospital. On day 9, his oxygenation suddenly showed a remarkable deterioration and he required 10 liters per minute of oxygen by face mask. An arterial gas analysis revealed that his PaO_2_ level was 50 mmHg. He received a tentative diagnosis of COVID-19-induced pneumonia accompanied by severe ARDS, and urgent tracheal intubation was performed under sedation by midazolam and rocuronium. Subsequently, he underwent mechanical ventilation with 1.0 of FiO_2_ and 10 cmH_2_O, with positive end-expiratory pressure (PEEP). The results of arterial gas analysis at that time were as follows: pH, 7.217; PaO_2_, 86.6 mmHg; PaCO_2_, 60.0 mmHg, and $\textrm{HCO}_{3}^{-}$, 23.5 mmol/L. He also received intravenous immunoglobulin (IVIG) and an infusion of glycyrrhizin. The immunoglobulin had been extracted from blood obtained from a Japanese donor. His clinical course with treatment and the results of a biochemical analysis are shown in Figure 2.

His condition was complicated by marked sinus bradycardia, which tended to decrease to under 30 beats per minute at night under sedation with midazolam. However, his systolic blood pressure remained >100 mmHg; thus, the bradycardia was only observed. His oxygenation gradually improved, with his PaO_2_/FiO_2_ value reaching near 300; thus, extubation was performed on day 15. After rehabilitation, he no longer required oxygen on day 19. As a treatment summary, he underwent 17 days of cefotaxime (2 g/day), 3 days of azithromycin (500 mg/day), 15 days of heparin (12,000-13,000 unit/day), 11 days of dexamethasone (6 mg/day), 10 days of remdisvir (100 mg/day), 3 days of IVIG (5 g/day), 7 days of glycyrrhizin (80 ml/day), and 4 days glycyrrhizin (40 ml/day). On day 20, his D-dimer (18 μg/mL) level was found to be increased and enhanced CT revealed pulmonary embolism. As his oxygenation did not change, a direct oral anticoagulant was prescribed. After twice confirming COVID-19-negativity by PCR, he was moved to a general ward on day 28. Marked sinus bradycardia was not observed at night. He was discharged on day 59 after rehabilitation.

## Discussion

COVID-19-induced pneumonia was thought to be the main cause of ARDS in the present case. Although antiviral therapy is available for some respiratory viral infections, most viruses do not have any specific treatment. Remdesivir was administered as an antiviral agent in the present case. Remdesivir is an intravenous nucleotide prodrug of an adenosine analog. Remdesivir binds to the viral RNA-dependent RNA polymerase, inhibiting viral replication through premature termination of RNA transcription. Activity against severe ARDS induced by SARS-CoV-2 has been demonstrated in vitro [4]. Clinically, remdesivir was superior to placebo in shortening the time to recovery in adults who were hospitalized with COVID-19 who had evidence of lower respiratory tract infection [5]. However, the present case developed ARDS even after the infusion of remdesivir. The other antiviral agent was glycyrrhizin. Glycyrrhiza glabra roots contain glycyrrhizic acid (glycyrrhizin), which is effective against viruses [6]. Glycyrrhizin inhibits the growth and cytopathology of several unrelated DNA and RNA viruses while not affecting human cell activity or the ability of human cells to replicate [6]. Glycyrrhizin is therefore currently applied in the treatment of various viral infections [6-10]. Traditional Chinese medicines including glycyrrhizin may also be effective against COVID-19 infection [11,12]. However, there have been no reports focusing on the efficacy of glycyrrhizin alone against COVID-19 infection because traditional Chinese medicines include not only glycyrrhizin but also a number of herbal medicine components. In addition, macrolide and IVIG can also exert an antiviral effect [13]. One report described that IVIG derived from healthy donors in Japan before the start of the COVID-19 pandemic had no direct effect against SARS-CoV-2 [14]. However, a randomized placebo-controlled double-blind clinical trial revealed that the administration of IVIG to patients with severe COVID-19 infection who did not respond to initial treatment could improve their clinical outcome and significantly reduce their mortality rate [15]. The difference in the dose of IVIG between the previous clinical trial (20 g/day) and the present case (5 g/day) was based on differences in the insurance system [15], as the Japanese medical insurance system does not allow for the administration of more than 5 g/day for 3 days for septic patients. General human strains of coronavirus have been associated with the common cold. Cross-reactivity between antibodies to different human coronaviruses has been confirmed [16]. Accordingly, antibodies to general human coronaviruses in IVIG may have an antiviral effect against SARS-CoV-2, even though IVIG lacks direct neutralizing and binding activities against SARS-CoV-2 in vitro. In addition, Nobel prize winner Dr. Shinya Yamanaka has postulated that Japanese individuals may possess an X-factor, which allows the Japanese population to experience low rates of death and infection from COVID-19. Candidates for such an X-factor include an activated immune system induced by Bacillus Calmette-Guérin vaccination or cross-reactivity for previous epidemic coronaviruses in Japan. The present patient was treated with immunoglobulin extracted from the blood that was obtained from a Japanese donor, the X-factor in the immunoglobulin may thus have played a role in the patient obtaining a favorable outcome (https://asia.nikkei.com/Business/Science/Yamanaka-on-COVID-19/Uncovering-Japan-s-coronavirus-X-factor-matters-to-the-world). These unspecific antiviral immune modulation therapies may be useful treatments for the main cause of ARDS, which may explain the favorable outcome obtained in the present case.

This is a unique case that may support the provision of alternative treatments involving the combination of IVIG and glycyrrhizin after the failure of initial standard treatments using heparin, dexamethasone, and remdisvir in COVID-19 patients. The treatment, in this case, was not part of the standard Japanese therapy, nor was it part of a randomized controlled trial. Treatment with IVIG and glycyrrhizin was added based on personal experience using this approach to treat a viral infection of unknown cause [9,10]. The timing was based on the fact that initial standard treatments for COVID-19 in Japan had failed; had the present patient received this combination of IVIG and glycyrrhizin as the initial treatment, he might not have developed ARDS and required mechanical ventilation. Further examinations are needed to confirm the efficacy of this approach, its indication, timing, duration, and optimal dose for COVID-19 infection.

The present case showed marked sinus bradycardia during treatment for COVID-19, and complicated pulmonary embolism during admission. A previous report showed the neuroinvasive potential of COVID-19 infection, which could result in damage of the cardiorespiratory center in the brain stem or alteration of the intrinsic cardiac nervous system, causing autonomic dysfunction, resulting in sinus node dysfunction [17]. Gubitosa et al. reported the case of a patient with marked sinus bradycardia that began acutely following the initiation of remdesivir and which resolved almost immediately upon cessation of the drug [18]. Remdesivir partly depends on its affinity for viral RNA polymerase rather than human mitochondrial RNA polymerase (h-mtRNAP). However, the possibility of h-mtRNAP involvement and subsequent mitochondrial dysfunction is still plausible, with drug-induced mitochondrial dysfunction resulting in cardiotoxicity. Accordingly, COVID-19 infection itself or a side effect of remdesivir may have caused the patient’s marked bradycardia. Venous thromboembolism is a well-known complication of COVID-19 infection [19,20]. Accordingly, in addition to early therapeutic anticoagulation for severe COVID-19 patients, careful decision-making in relation to the timing of cessation of therapeutic anticoagulation (when the patient is able to walk without assistance) is important.

## Conclusions

Glycyrrhizin inhibits the growth and cytopathology of several unrelated DNA and RNA viruses while not affecting human cell activity or the ability of human cells to replicate. General human strains of coronavirus have been associated with the common cold. Cross-reactivity between antibodies to different human coronaviruses has been confirmed, and the administration of IVIG to patients with severe COVID-19 infection may improve their clinical outcomes. These unspecific antiviral therapies and immune modulation therapy may be useful treatments for the main cause of ARDS, which may explain the favorable outcome that was obtained in the present case.
